# The Aboveground Biomass Estimation of the Grain for Green Program Stands Using UAV-LiDAR and Sentinel-2 Data

**DOI:** 10.3390/s25092707

**Published:** 2025-04-24

**Authors:** Gaoke Yueliang, Gentana Ge, Xiaosong Li, Cuicui Ji, Tiancan Wang, Tong Shen, Yubo Zhi, Chaochao Chen, Licheng Zhao

**Affiliations:** 1 School of Smart City, Chongqing Jiaotong University, Chongqing 400074, China; yuelianggaoke@163.com (G.Y.); cuicuiji@whu.edu.cn (C.J.); 2 Aerospace Information Research Institute, Chinese Academy of Sciences, Beijing 100094, China; wangtiancan22@mails.ucas.ac.cn (T.W.); shentong@aircas.ac.cn (T.S.); zyb5289@gmail.com (Y.Z.); chenchaochao23@mails.ucas.ac.cn (C.C.); zhaolicheng@aircas.ac.cn (L.Z.); 3 Forestry and Grassland Work Station of Inner Mongolia, Hohhot 010010, China; nmgtghl@126.com

**Keywords:** aboveground biomass, UAV-LiDAR, grain for green in Inner Mongolia, sustainable forest management

## Abstract

Aboveground biomass (AGB) serves as a crucial indicator of the effectiveness of the Grain for Green Program (GGP), and its accurate estimation is essential for evaluating forest health and carbon sink capacity. However, due to the dominance of sparse forests in GGP stands, research in this area remains significantly limited. In this study, we developed the optimal tree height-diameter at breast height (DBH) growth models for major tree species and constructed a high-quality AGB sample dataset by integrating airborne LiDAR data and tree species information. Then, the AGB of the GGP stands was estimated using the Sentinel-2 data and the gradient boosting decision tree (GBDT) algorithm. The results showed that the AGB sample dataset constructed using the proposed approach exhibited strong consistency with field-measured data (R^2^ = 0.89). The GBDT-based AGB estimation model shows high accuracy, with an R^2^ of 0.96 and a root mean square error (RMSE) of 560 g/m^2^. Key variables such as tasseled cap greenness (TCG), red-edge normalized difference vegetation index (RENDVI), and visible-band difference vegetation index (VDVI) were identified as highly important. This highlights that vegetation indices and tasseled cap transformation index information are key factors in estimating AGB. The AGB of major tree species in the new round of the GGP stands in Inner Mongolia ranged from 120 to 9253 g/m^2^, with mean values of 978 g/m^2^ for poplar, 622 g/m^2^ for Mongolian Scots pine, and 313 g/m^2^ for Chinese red pine species. This study offers a practical method for AGB estimation in GGP stands, contributing significantly to sustainable forest management and ecological conservation efforts.

## 1. Introduction

Ecological restoration has emerged as a critical strategy for reversing land degradation and enhancing ecosystem resilience, with China placing significant emphasis on this approach to tackle its pressing environmental challenges. China has implemented a series of large-scale afforestation initiatives, among which the Grain for Green Program (GGP) is a significant ecological restoration initiative returning the slope cropland to forests or grasslands [1,2]. Currently, the GGP has entered a new phase, with the second round of subsidy policies set to expire progressively starting in 2022. However, the forests and vegetation established under the program remain unstable, and the economic benefits are not yet evident, necessitating urgent efforts to consolidate the achievements of farmland restoration. Inner Mongolia is the province with the largest area of farmland converted to forests and grasslands under the GGP, with a cumulative total of 3.03 million hectares by the end of 2019 [3]. As of now, this figure has increased to 3.12 million hectares, primarily focused on forests. The project area spans a vast east–west range, with the widespread use of tree and shrub species such as Mongolian Scots pine (*Pinus sylvestris* var. *mongholica*), poplar (*Populus* spp.), and korshinsk peashrub (*Caragana korshinskii*). Aboveground biomass (AGB) is a key indicator of the GGP’s effectiveness, which not only reflects the growth status of vegetation but is also closely linked to the carbon stock and ecosystem services of forested areas [4]. Therefore, accurately estimating the AGB of GGP stands is essential not only for evaluating the ecological benefits of the program but also for providing critical data to support China’s carbon sink monitoring.

Destructive sampling is the most accurate and reliable method for measuring AGB [5]. However, its extensive application is impractical due to the significant destruction of forests that it entails, as well as its time-consuming, labor-intensive, and costly nature [6]. With advancements in remote sensing technology, remote sensing has become a widely used tool for estimating forest canopy structure and AGB, as it can provide accurate and rapid vegetation cover information over large areas [7]. Among these technologies, light detection and ranging (LiDAR), as an active remote sensing method, has demonstrated strong penetration capabilities, allowing for the rapid and accurate acquisition of three-dimensional vegetation data, as well as horizontal and vertical structural information on forest canopies [8]. The point cloud data obtained from LiDAR can be used to derive precise structural attributes such as tree height, DBH, canopy size, and more [9,10], making it an effective tool for forest AGB estimation. The integration of LiDAR-derived structural attributes with other remote sensing data has become a prominent approach for estimating AGB across various forest ecosystems. Studies combining LiDAR with optical satellite data, such as Sentinel-2 and Landsat, have utilized UAV or airborne LiDAR’s high-resolution canopy data alongside the extensive spatial coverage of optical imagery to monitor forest restoration and large-scale biomass distribution [11,12]. Similarly, pairing LiDAR with hyperspectral data has enabled the detailed mapping of AGB at individual tree and regional scales by merging structural information with vegetation spectral signatures [13,14]. Furthermore, integrating LiDAR with synthetic aperture radar (SAR) data, such as Sentinel-1, has improved AGB estimates in complex environments by leveraging radar’s sensitivity to forest structure [15].

Currently, the estimation of the aboveground biomass of forests mainly focuses on typical forests with dense canopy cover and tall trees [16,17]. However, GGP stands possess unique characteristics that distinguish them from such forests. Taking the new round of GGP in Inner Mongolia as an example, which began implementation in 2015, these stands are characterized by younger tree age, lower canopy cover, and relatively shorter tree height. These characteristics present significant challenges for large-scale AGB estimation with remote sensing technology, and there is currently a lack of targeted research in this specific area. Airborne LiDAR data offer a promising solution, as they can efficiently and accurately extract tree attributes such as height and crown width from point cloud data, which are closely related to AGB estimations [18]. Moreover, LiDAR could serve as a critical bridge between ground-based measurements and satellite imagery [13,19]. Sentinel-2 imagery provides data with continuous spatial coverage, which, in combination with LiDAR, enables wide-area AGB estimation. Building on this, the present study developed optimal tree height–DBH growth models for major tree species based on measured individual tree data. Using these models, along with biomass equations, a high-quality AGB sample dataset was constructed by integrating LiDAR data and tree species information. Furthermore, Sentinel-2 data, combined with machine learning models, were utilized to explore the feasibility of AGB estimation in GGP stands.

## 2. Materials

### 2.1. Study Area

**Figure 1:**
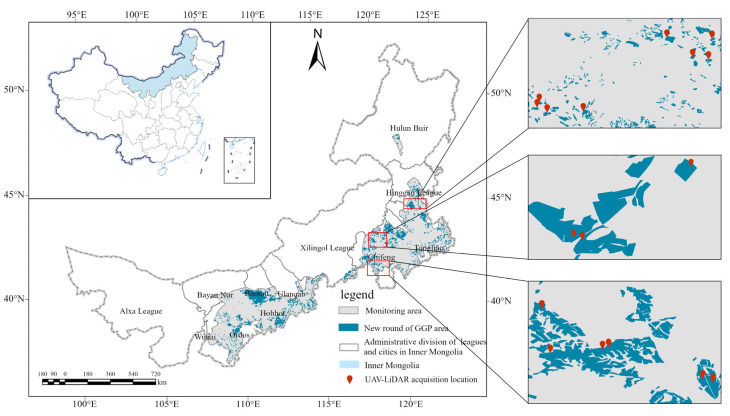
An overview map of the study area.

Inner Mongolia is located along the northern border of China (Figure 1). The region transitions gradually from humid and semi-humid zones in the northeast to semi-arid and arid zones in the southwest. Temperature and precipitation exhibit significant spatial and temporal variability across the region. The geomorphology is complex and diverse, dominated by plateaus with an average elevation exceeding 1000 m. Additionally, due to its unique geographic location, topography, and climatic conditions, the ecological environment in Inner Mongolia is highly fragile. The region faces severe challenges such as grassland degradation, soil erosion, and the uneven distribution of water resources [20,21,22,23,24].

Inner Mongolia began implementing a new round of the GGP in 2015, initiating planting operations. By 2022, the program encompasses more than 110,000 stands, with a total fallow land area of 202,700 ha. The stands span a wide east–west range, resulting in significant variability in regional climate and soil conditions. Consequently, the types of vegetation configurations and growth conditions in the fallow fields also exhibit considerable differences. Overall, shrubs dominate the fallow forest area, covering approximately 140,000 ha and accounting for 67.4% of the total. Tree forests rank second, with an area of about 50,000 ha, representing 25.5% of the total. Mixed tree–shrub forests occupy the smallest area, around 10,000 ha, making up 7.1% of the total. The primary tree species planted include Mongolian Scots pine, poplar, Chinese red pine (*Pinus tabuliformis*), and korshinsk peashrub.

### 2.2. Data Acquisition and Preprocessing

#### 2.2.1. Sentinel-2 Data

The Sentinel-2 satellite is a next-generation Earth observation system developed and launched by the European Space Agency (ESA). It consists of two identical satellites, Sentinel-2A and Sentinel-2B, which were successfully launched on 23 June 2015 and 7 March 2017, respectively, forming a dual-satellite constellation. Each satellite is equipped with an advanced multispectral imager capable of capturing data across 13 spectral bands at spatial resolutions of 10 m, 20 m, and 60 m. This system supports a wide range of applications, particularly in Earth observation and environmental monitoring. In this study, the zenith reflectance dataset (Product ID: COPERNICUS/S2_SR) available on the Google Earth Engine (GEE) platform was utilized. Image data from the period of 1 June to 1 September 2023 were selected for vegetation index calculations. This zenith reflectance dataset is a Level-2A product that has undergone topographic corrections using a digital elevation model (DEM) and atmospheric correction via the sen2cor tool. As a result, the dataset is pre-processed and does not require additional image correction, ensuring reliable and ready-to-use data for analysis.

#### 2.2.2. UAV-LiDAR Data

In this study, airborne LiDAR data collection was conducted in July 2024 at three locations: Horqin Right Wing Middle Banner, Songshan District, and Linxi County. A total of 30 sets of high-precision UAV LIDAR point cloud data were collected in this survey, covering an area of about 370 hectares. The main tree species collected were poplar, camphor pine, and oil pine, of which poplar comprised about 215 hectares, Mongolian Scots pine 50 hectares, and Chinese red pine 105 hectares. All data were acquired using the Zenith L1 sensor mounted on DJI’s (Guangzhou, China) Longitude M300 RTK drone. The size of the plot areas and the specific characteristics of the UAV, particularly its payload capacity and resulting flight time, were carefully considered to determine the coverage area for each flight and the number of flights required to cover the entire region. The flights were carried out at an altitude of 60/80 m, with a side overlap of no less than 60% and a forward overlap of 85%. A triple echo pattern was employed, resulting in a point cloud density of approximately 450 points per square meter.

The UAV data were post-processed using the 3D reconstruction module of DJI Terra to handle the LiDAR point cloud data. The point cloud fusion parameter was set to “high density” (100% sampling rate), ensuring that all point cloud data were utilized in processing. This setting produces the highest quality results but requires the longest processing time. Additionally, point cloud accuracy optimization was enabled, which refines the point cloud data collected at different time intervals, thereby enhancing the overall accuracy of the final dataset. Subsequently, point cloud data were pre-processed using LiDAR 360 software (Version 5.2), which included steps such as denoising, classification, and normalization. During the classification process, the improved progressive TIN densification (IPTD) filtering algorithm [25] was employed to categorize denoised data into ground points, vegetation points, and building points.

#### 2.2.3. Field Survey Data

The ground survey data for this study were collected from representative GGP stands in Inner Mongolia, with sample plots established across six county-level administrative regions: Horqin Right Wing Middle Banner, Duolun County, Linxi County, Songshan District, Qingshuihe County, and Jungar Banner. A total of 79 sample plots were surveyed during 2023 and 2024, and the major tree species of GGP stands were poplar, Mongolian Scots pine, and Chinese red pine. In this study, we selected a homogeneous forest plot with an area larger than 120 m × 120 m located in the fallow forest plot, and we laid a 30 m sample line in a due north direction and two other 30 m sample lines at 60° intervals, with the midpoints of the three sample lines intersecting at the center of the sample plot. A series of parameters were recorded during the field survey, including the RTK coordinates of the sample centers; geographic information such as elevation; and the diameter at breast height (DBH) of individual trees, tree height, crown width, and the number of trees in the sample plots.

To estimate the aboveground biomass of the sample plots, this study employed the biomass models for poplar, Mongolian Scots pine, and Chinese red pine outlined in the national standard “Tree biomass models and related parameters to carbon accounting for major tree species” (GB/T 43648-2024) [26], developed under the guidance of the National Forestry and Grassland Administration. The equations used for the aboveground biomass estimation are presented in Table 1.

Here, Ma represents the AGB of individual trees, D denotes the diameter at breast height, and H refers to tree height. To calculate the AGB, different models were applied based on the distribution of tree species within each sample plot. Using the above equations, the biomass of individual trees for each species was calculated and then multiplied by the number of trees in the corresponding sample plot. This approach allowed for the determination of the biomass distribution within each plot. Finally, the total AGB of each sample plot was standardized and expressed in grams per square meter (g/m^2^).

#### 2.2.4. SRTM DEM Data

The Shuttle Radar Topography Mission (SRTM) was a collaborative project involving NASA, the U.S. Department of Defense, and the German and Italian space agencies. The mission collected satellite data over a 10-day period from 11 February to 22 February 2000, producing two types of digital elevation models with spatial resolutions of 30 m and 90 m. In this study, 30 m resolution SRTM DEM data were utilized. The SRTM data were employed to extract key topographic parameters, including ground elevation, slope, and slope aspect, which were used as input variables in the model to account for and mitigate the influence of topography on AGB estimations. These data are also available in the GEE platform.

#### 2.2.5. The GGP Stands Distribution Data

The stand distribution data used in this study were provided by the General Station of Forestry and Grassland Work of Inner Mongolia. The data were organized as a vector database on a county-level basis, containing detailed geographic information, including the location and boundaries of stands. Additionally, the database included key attribute information such as the year of implementation, the main tree species, and the current land cover. The dataset comprises a total of 110,229 GGP stands, including 36,084 tree forest stands, 65,321 shrub forest stands, and 8824 mixed tree–shrub forest stands. The operational period covered by the data spans eight years, beginning in 2015 and concluding in 2022. These data provide the basis for the selection of geographic locations for the collection of measured data and LiDAR data, and they provide tree species information for the subsequent construction of the aboveground biomass sample set.

## 3. Methods

**Figure 2:**
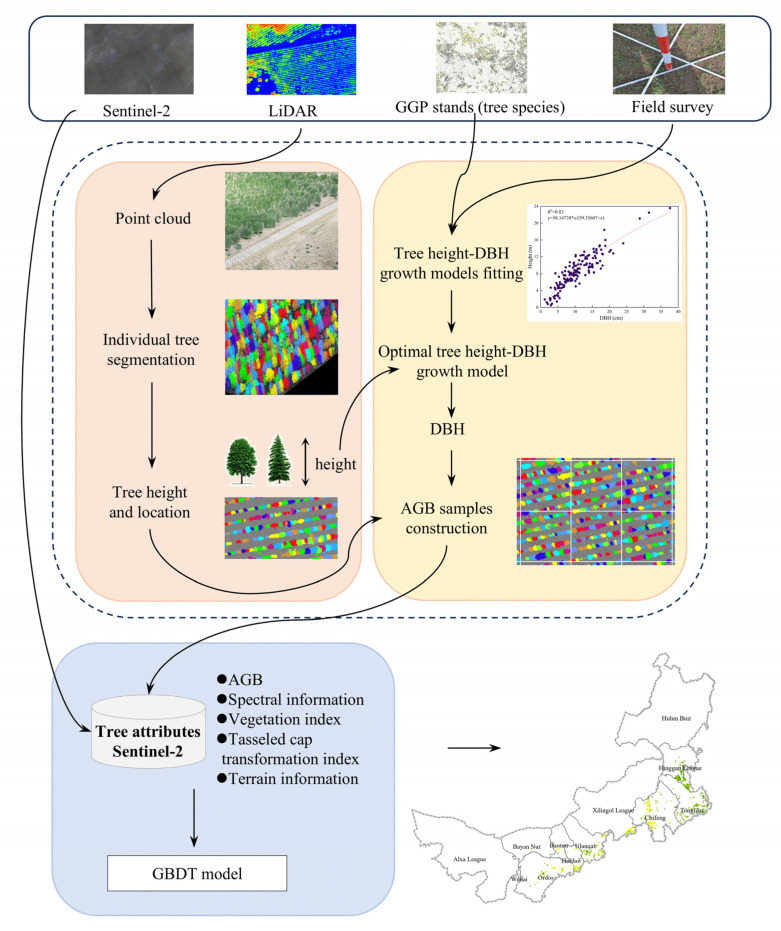
Overall methodology flowchart.

The methodology of this study is divided into two main parts, as shown in Figure 2. One part focuses on the construction of AGB samples, while the second part involves the estimation of AGB using machine learning methods.

### 3.1. Construction of AGB Samples Based on LiDAR Data

First, this study utilized LiDAR data to obtain individual tree heights through an individual tree segmentation algorithm. Using field survey data, tree height–DBH growth models were fitted for major tree species, and the optimal model was applied to estimate individual tree DBH by integrating LiDAR-derived parameters (e.g., tree height and location) with species information from GGP stand distribution data. Second, species-specific biomass calculation equations were employed to calculate the individual tree AGB, and a grid-based sampling approach was used to spatially partition LiDAR data, constructing a high-quality AGB sample set.

#### 3.1.1. Individual Tree Segmentation

In this study, individual tree segmentation is used to extract individual tree height and location parameters, where individual tree segmentation is implemented using a point cloud segmentation algorithm [27], and tree attributes such as crown diameter, area, and volume can be extracted at the same time. In the specific implementation process, the algorithm first scans the entire point cloud data to find the global elevation maxima as the seed point A, which usually corresponds to the vertex position of the tree crown. Subsequently, according to the preset spacing threshold and minimum spacing rule, the algorithm takes seed point A as the starting point and gradually expands downward by area growth to cluster eligible 3D point cloud data into single-wood point cloud clusters. Among them, the spacing threshold is a key parameter, which directly affects the accuracy of segmentation, and this study combines the average crown diameter obtained from field sampling with dynamic adjustment to adapt to the differences in different tree species and stand conditions. After completing single-tree segmentation, the algorithm can further extract single-tree structural attribute parameters such as location coordinates, tree height, crown diameter, crown area, crown volume, and so on. These parameters can reflect the growth status of single trees and provide important data support for the subsequent construction of the aboveground biomass sample set.

#### 3.1.2. DBH Acquisition

To obtain the DBH of individual trees from airborne LiDAR data, this study estimated DBH using an optimal tree height–DBH growth model fitted with field survey data. Drawing on the domestic and international studies of tree height–DBH growth models, we utilized field-measured individual tree data to fit four commonly used models (Table 2) based on the overall trend observed in tree height–DBH scatterplots for the dominant tree species. Through a comparative analysis of the fitting performance of these models, the best tree height–DBH growth models were selected for poplar, Mongolian Scots pine, and Chinese red pine in the GGP region. Subsequently, tree heights obtained from individual tree segmentation were used to select the corresponding species-specific growth models for DBH estimation. The acquisition of DBH aims to provide data support for the subsequent construction of AGB samples based on airborne LiDAR data.

#### 3.1.3. Construction of AGB Samples

Following individual tree segmentation, matching each tree to a specific species is a crucial step. To achieve this, tree coordinates derived from point cloud data segmentation were integrated with GGP stand data and field sampling data that provide tree species information. This process links the structural attributes of trees (height and crown width) with their biological attributes (species). The study area predominantly consists of monoculture plantation forests, with each plot containing a single tree species. By spatially overlaying point cloud data with GGP stands data, the tree species information corresponding to each point cloud dataset was identified. Each species exhibits different growth patterns and biomass relationships, and the integration of tree coordinates with species information ensures that each tree is associated with its corresponding species, which is critical for subsequent accurate AGB estimation using tree height–DBH growth models and species-specific AGB equations.

In this study, point cloud data from individual tree segmentation were gridded at a 30 m resolution. Based on species-specific information, the optimal tree height–DBH growth model was selected for each tree species. Tree heights derived from LiDAR data were then used to calculate the DBH of each tree. Subsequently, the AGB of individual trees was estimated using the species-specific AGB models listed in Table 1. Finally, the total AGB for each grid was computed by summing the AGB of all individual trees within that grid. The AGB of each sample was calculated using Equation (1) and expressed in grams per square meter (g/m^2^). To ensure the uniform distribution of vegetation within the sample and reduce the influence of mixed information, samples located at the edge of the stand, as well as near roads and buildings, were removed.

(1) $$\mathrm{AGB}=\sum_{i=1}^{n}{\mathrm{Ma}}_{i}/S$$

Here, AGB is the sample aboveground biomass, Ma is the individual tree aboveground biomass, S is the grid area, and i is each tree.

### 3.2. Extracting Key Variables for AGB Estimation

The variables used could be categorized into 4 classes, namely, spectral information, vegetation index, tasseled cap transformation index, and terrain information. Vegetation indices are simple yet effective metrics for assessing surface vegetation conditions, calculated by leveraging the ratio or difference between specific spectral bands. These indices reflect the spectral characteristics of vegetation, exhibiting high sensitivity to vegetation cover and growth vigor and providing quantitative information on vegetation growth status, spatial distribution, and temporal changes. In order to reduce the impact of soil background and enhance the sensitivity of sparse vegetation areas to vegetation, this study selected vegetation indices such as SAVI and MASVI to improve the impact of the soil background. In this study, pre-processed image data from Google Earth Engine (GEE) were utilized, and all bands were resampled to a spatial resolution of 30 m using the nearest neighbor interpolation method [28]. A total of 26 variables, including NDVI and SAVI, were calculated for model construction, with the results stored in the GEE cloud-based dataset. To ensure consistency in spatial resolution, all characterization variables—such as Sentinel-2 spectral information, vegetation index, tasseled cap transformation index, and terrain information—were standardized to a resolution of 30 m. Variable-specific information is provided in Table 3.

### 3.3. GBDT Model for Inverting AGB

The gradient boosting decision tree algorithm is a highly effective machine learning model that constructs a series of weak learners and aggregates their predictions to produce the final output. By using the negative gradient of the loss function to fit residuals and iteratively correcting these residuals, the model progressively enhances its predictive performance. This approach offers high accuracy, robustness, and flexibility, making GBDT suitable for both regression and classification tasks. In this study, 80% of the samples were randomly selected as the training set, while the remaining 20% were used as the validation set. The eight bands and each pixel in the Sentinel-2 data corresponding to the vegetation index, tasseled cap transformation index, terrain factor and canopy cover, and tree height were input as explanatory variables in the model, and the AGB was used as the estimation variable. The model was implemented on the Google Earth Engine cloud platform using the gradient boosting regression tree algorithm. After verifying the model’s accuracy using the validation set, the trained model was applied to each pixel in the Sentinel-2 imagery. This process would output the estimated aboveground biomass value of each pixel so as to obtain the GGP stand AGB of major tree species.

### 3.4. Accuracy Evaluation

In this study, the coefficient of determination (R^2^), the root mean square error (RMSE), the sum of squares due to error (SSE) and Akaike information criterion (AIC) are used as the evaluation indexes of the model R^2^, reflecting the degree of explanation of the independent variable relative to the dependent variable in the regression prediction model and R^2^∈[−1, 1]. The larger R^2^ represents a better model fit and larger degree of explanation of the independent variable relative to the dependent variable. RMSE represents the deviation of the predicted value from the true value, and the larger RMSE represents the predicted value with a larger deviation from the true value. RMSE indicates the deviation of the predicted value from the true value; the larger the RMSE, the larger the deviation of the predicted value from the true value. SSE represents the sum of the squared errors between the predicted values and the true values; the closer SSE is to 0, the better the model selection and fitting. A smaller AIC means a better model fit.

(2) $$R^{2}=1-\sum_{i=1}^{n}\frac{{\left(P_{i}{-O}_{i}\right)}^{2}}{{\left(O_{i}{-Q}_{i}\right)}^{2}}$$

(3) $$\mathrm{RMSE}=\sqrt{\frac{\sum_{i=1}^{n}{\left(P_{i}{-O}_{i}\right)}^{2}}{n}}$$

(4) $$\mathrm{SSE}={\sum_{i=1}^{n}\left(P_{i}{-O}_{i}\right)}^{2}$$

(5) AIC=−2ln(L)+2k

Here, n is the number of samples, P represents the predicted tree height value, O represents the true value of tree height, and Q is the average of the true values. L is the maximum likelihood under the model, and k is the number of variables in the model.

## 4. Results

### 4.1. DBH Acquisition

**Figure 3:**
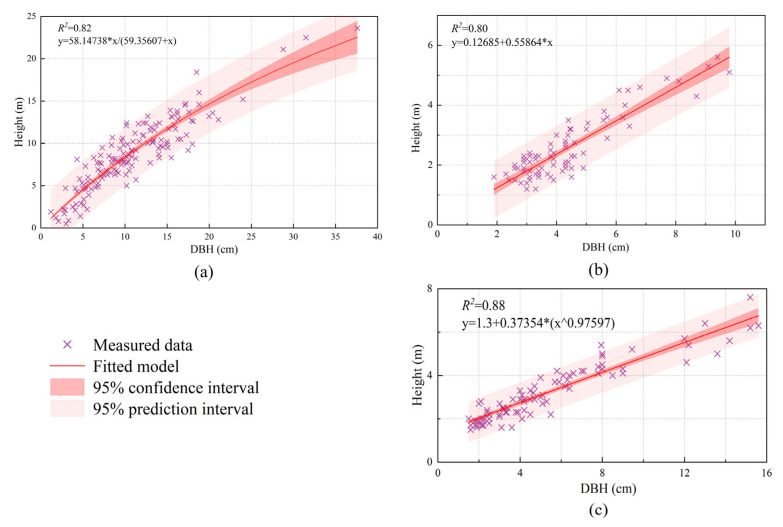
Optimal tree height–DBH growth models: (**a**) poplar; (**b**) Mongolian Scots pine; (**c**) Chinese red pine.

This study utilized tree heights obtained from individual tree segmentation to select the optimal growth model for each corresponding species to estimate DBH. Using the growth models listed in Table 2, we fitted and analyzed field-surveyed individual tree height and DBH data for three major species (Table 4). The performance metrics of the models showed minimal differences across the dominant species; thus, the tree height–DBH growth model with the best evaluation metrics, combined with tree heights derived from LiDAR data, was applied to estimate the DBH of individual trees for each species within the LiDAR data. Figure 3 illustrates the optimal tree height–DBH growth models identified for the three major tree species.

### 4.2. Construction of AGB Samples

**Figure 4:**
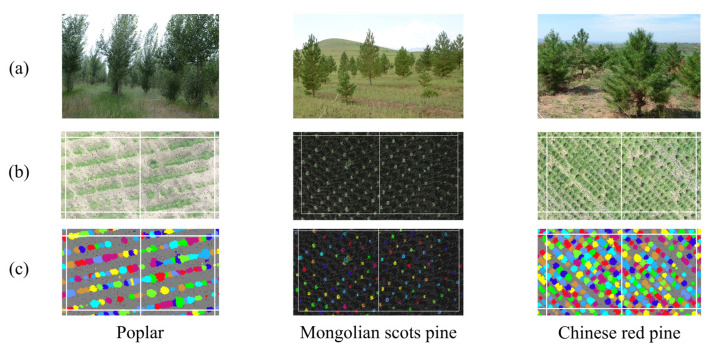
Example of AGB sample construction: (**a**) photos of measured sample plots; (**b**) LiDAR point cloud data; (**c**) individual tree segmentation. Different colors in (**c**) represent a separate tree.

After obtaining the tree height and DBH of individual trees from LiDAR data, species-specific biomass equations listed in Table 1 were applied to calculate the aboveground biomass (AGB) of individual trees. Subsequently, a grid-based sampling method was employed to spatially partition the LiDAR data, forming an AGB sample dataset, from which a total of 720 valid samples were selected in this study (Figure 4). The sample set includes 300 samples of poplar, 130 samples of Mongolian Scots pine, and 290 samples of Chinese red pine. These samples were used for the subsequent development of the AGB estimation model. The biomass values in the sample set range from 22 to 13,053 g/m^2^, with a mean of 1373 g/m^2^. Notably, 75% of the samples are distributed below 5000 g/m^2^, while biomass values between 5000 and 8000 g/m^2^ are relatively scarce. This distribution is closely related to the characteristics of the study area, which is characterized by young forest stands and low canopy cover.

**Figure 5:**
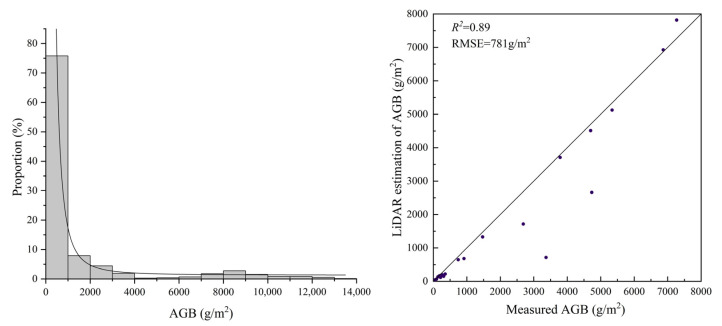
AGB sample set information (**left**) and validation result (**right**).

In order to further prove the consistency of this sample set with respect to the field data, this study validates the sample set with the field sample points, with a total of 21 overlapping points. Moreover, the validation results are shown in Figure 5, which shows that the AGB sample set constructed in this study using LiDAR point cloud data has better consistency with respect to the field data. The validation points are mostly concentrated near the 1:1 line, and the validation accuracy is R^2^ = 0.89 and RMSE = 781 g/m^2^. This shows that the sample set constructed in this study can replace the field sample points as the ground truth to participate in the model’s construction.

### 4.3. GBDT Model Construction and Accuracy Analysis

#### 4.3.1. GBDT Model Parameter Optimization

During the parameter tuning process of the gradient boosting decision tree model, the primary focus was on adjusting the number of weak learners (ntree) and the learning rate (shrinkage). The learning rate determines the contribution of each decision tree to the final prediction and is closely related to the number of decision trees. In this study, the learning rate was evaluated within the range of [0.005, 0.010, 0.050, 0.001], while the number of decision trees (ntree) was varied from 100 to 800 with a step size of 100. In order to determine the optimal parameter combinations, a grid search method was used in this study, and the R^2^ values on the test set were used as an error criterion for optimal parameter determination. The results demonstrated that the R^2^ value increased progressively with the growth of ntree, plateauing once the optimal parameter combination was reached. Following the evaluation of the regression model, the optimal parameters were determined to be an ntree value of 300 and a learning rate of 0.01.

**Figure 6:**
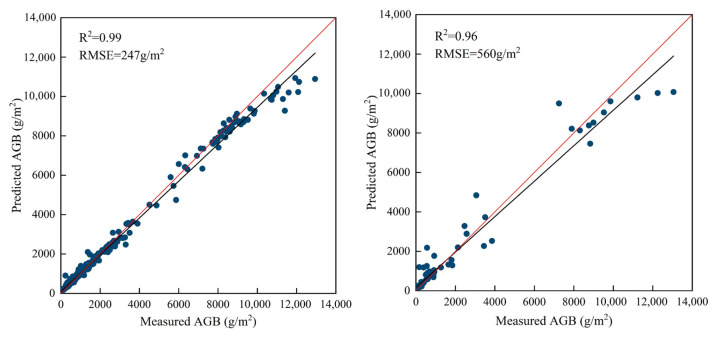
Plots of model training (**left**) and validation (**right**) fitting results.

Based on the optimal parameters, this study utilized the gradient boosting decision tree regression algorithm to construct the AGB estimation model, and the results of model training and validation are shown in Figure 6, with a model training accuracy of R^2^ = 0.99 and RMSE = 247 g/m^2^ and a model validation accuracy of R^2^ = 0.96 and RMSE = 560 g/m^2^, which showed that the overall results of the AGB estimation model are good.

#### 4.3.2. Variable Importance Analysis

**Figure 7:**
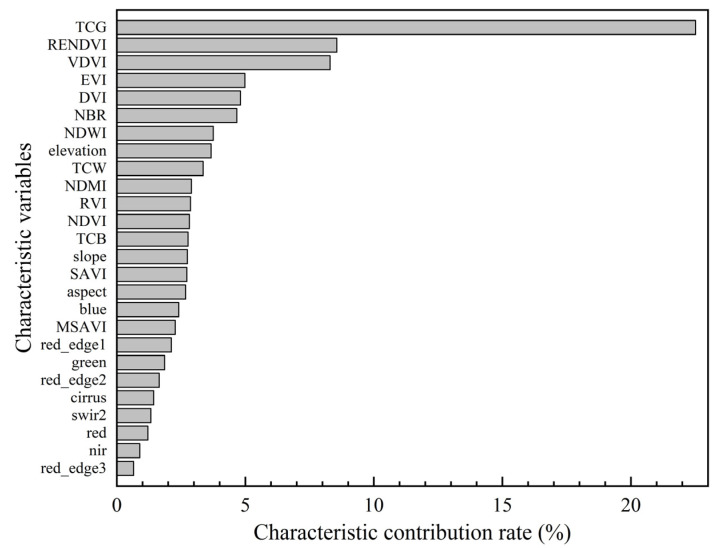
Ranking of the model’s characteristic variable contributions.

In this study, a total of 26 variables were derived from optical imagery, terrain data, and auxiliary datasets. Using the gradient boosting decision tree regression algorithm, the contribution rates of each variable to the AGB estimation model were ranked based on their importance. The results, as presented in Figure 7, indicate that the three most influential variables are “TCG”, “RENDVI”, and “VDVI”. This highlights the significant role of the tasseled cap transformation index and vegetation indices in capturing critical spatial variations in AGB. Among these, the contribution rate of the tasseled cap transformation index far surpasses the other variables, underscoring its pivotal role in the biomass estimation model. This phenomenon can likely be attributed to the fact that the tasseled cap transformation incorporates critical structural and compositional information. It effectively reduces the influence of soil brightness in the imagery while enhancing the greenness component. This effect becomes increasingly pronounced with greater vegetation coverage, as the transformation accentuates the spectral differences associated with varying levels of vegetation density.

### 4.4. AGB Distribution of Major Tree Species

**Figure 8:**
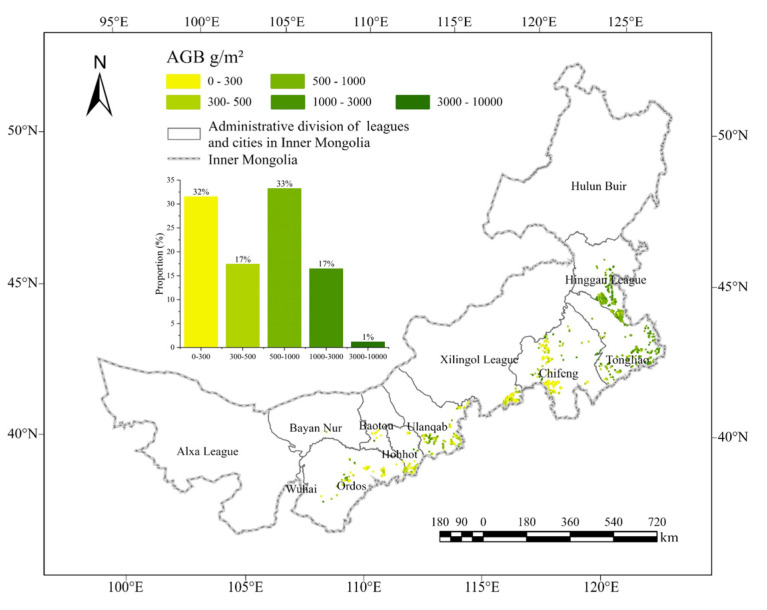
AGB distribution map of the main tree species in GGP stands.

This study obtained the AGB distribution map for the main tree species in the GGP stands based on the AGB estimation model. The statistics reveal that the estimated AGB (Figure 8) for the major tree species in the new round of GGP in Inner Mongolia ranges from 120 to 9253 g/m^2^. Among these, there are a total of 5484 poplar stands, with AGB ranging from 137 g/m^2^ to 9232 g/m^2^ and an average value of 978 g/m^2^; 4640 Mongolian Scots pine stands, with AGB ranging from 120 g/m^2^ to 9253 g/m^2^ and an average value of 622 g/m^2^; and 3927 Chinese red pine stands, with AGB ranging from 127 g/m^2^ to 3861 g/m^2^ and an average value of 313 g/m^2^. The distribution of GGP stands with major tree species in Inner Mongolia spans a wide range, and it is primarily divided into eastern and western regions. The eastern region contains a higher number of planted plots, totaling 10,074, with an average AGB of 757 g/m^2^, while the western region has fewer plots, totaling 3979, with an average AGB of 465 g/m^2^.

## 5. Discussion

The GGP and other large-scale afforestation programs play a pivotal role in mitigating climate change, preserving biodiversity, and preventing land degradation. However, in the early stages of these projects, planted forests often feature low canopy densities due to immature tree growth, failing to form dense forest stands. This makes it challenging for satellite remote sensing to effectively monitor these areas [29]. The advancement of UAV LiDAR technology offers new opportunities for large-scale AGB estimation [30]. A few studies have demonstrated the feasibility of using UAV LiDAR data to map AGB distributions in sparse woodlands in arid and semi-arid regions [31,32]. This study focuses on AGB estimation in the GGP stands, leveraging LiDAR data, remote sensing imagery, and machine learning models. From AGB sample set construction to model validation, the proposed approach achieved satisfactory accuracies. The findings highlight the capability of UAVs combined with remote sensing imagery to effectively monitor AGB in sparse woodlands with low tree heights and low canopy densities. Additionally, this method addresses the challenge of limited ground-based sample data across large areas, laying a solid foundation for the long-term dynamic monitoring of GGP stands. However, the GGP stands are generally fragmented, and the spatial resolution of Sentinel-2 is still limited in capturing the subtle variability of AGB in sparsely vegetated areas of GGP; the introduction of higher-resolution remote sensing data in the future can further enhance the ability to estimate aboveground biomass, such as the combination of multispectral and UAV-LiDAR [33] or the combination of GF-1 imagery data and UAV-LiDAR to estimate AGB [34].

Spectral information from Sentinel-2, vegetation indices, and tasseled cap transformation indices was selected due to its ability to capture vegetation health and biomass variability. In the variable importance ranking of the AGB estimation model, “TCG”, “RENDVI”, and “VDVI” exhibited relatively high contributions. These findings were consistent with previous studies for sparse vegetation in arid and semiarid areas. Yan et al. [35] utilized tasseled cap transformation indices to estimate AGB, further confirming the relevance of greenness to biomass estimation. In sparse vegetation areas, spectral signals of vegetation greenness are typically weak, making them difficult to capture using traditional vegetation indices such as NDVI [36,37,38]. In arid and semi-arid regions, TCG effectively extracts the localized greenness features of sparse vegetation, distinguishing subtle differences between low-density vegetation and bare soil and thereby enhancing the recognition of sparse vegetation distributions [39]. Currently, the red-edge normalized difference vegetation index (RENDVI) is extensively utilized for vegetation detection and ecological monitoring in arid regions [40,41]. The visible-band difference vegetation index (VDVI) is highly sensitive to vegetation information and has been widely applied in arid desert regions with sparse vegetation, significantly improving vegetation detection and monitoring [42,43,44]. Clevers et al. [45] demonstrated the importance of Sentinel-2’s red-edge bands for vegetation estimation.

## 6. Conclusions

This study focused on the characteristics of sparsely distributed forest land in the new round of GGP stands in Inner Mongolia. We developed the optimal tree height–diameter at breast height growth models for the major tree species and constructed a high-quality AGB sample dataset by integrating airborne LiDAR data and tree species information. With the assistance of Sentinel-2 images and a gradient boosting regression tree model, the AGB of major tree species in these areas was estimated. The study draws the following conclusions:

(1) The high-quality AGB sample set constructed using LiDAR data and optimal growth models showed excellent agreement with field-measured data, making it a reliable substitute for traditional ground-based measurements in model development.
(2) This study used the AGB sample set, combined with Sentinel-2 images, to establish an AGB estimation model for the main tree species in the GGP stands. This can obtain the AGB of the main tree species in the GGP stands, with an R^2^ of 0.96 and an RMSE of 560 g/m^2^, which can provide reference and support for the evaluation of the effectiveness of GGP and ecological benefits.
(3) The tasseled cap transformation index (TCG) and vegetation indices such as RENDVI and VDVI were identified as key variables sensitive to the AGB estimation model. These variables have been demonstrated to be effective for extracting vegetation information in arid and semi-arid regions.
(4) The AGB of the major tree species in the new round of GGP stands in Inner Mongolia ranged from 120 to 9253 g/m^2^. The mean AGB of poplar was 978 g/m^2^, the mean AGB of Mongolian Scots pine was 622 g/m^2^, and the mean AGB of Chinese red pine was 313 g/m^2^. This distribution characteristic provides a reference for the follow-up conservation and management of the GGP.

## Figures and Tables

**Table 1:** Biomass calculation equations of major tree species in Inner Mongolia.

Tree Species	D < 5 cm	D ≥ 5 cm	R^2^	MPE (%)
Poplar	$\mathrm{Ma}=0.09585\times D^{1.67005}H^{0.59163}$	$\mathrm{Ma}=0.05559\times D^{2.00861}H^{0.59163}$	0.95	4.23
Mongolian Scots pine	$\mathrm{Ma}=0.17577\times D^{1.50770}H^{0.34775}$	$\mathrm{Ma}=0.05460\times D^{2.23412}H^{0.34775}$	0.94	5.10
Chinese red pine	$\mathrm{Ma}=0.39835\times D^{1.07994}H^{0.43610}$	$\mathrm{Ma}=0.067765\times D^{2.18050}H^{0.43610}$	0.94	5.12

**Table 2:** Tree height–DBH growth models.

Model Number	Model	Formula
1	Linear regression	H=a+b×DBH
2	Power function	H=1.3+a×DBH^b
3	Logarithmic model	H=a+b×lnDBH
4	Bates model	H=a×DBH/(b+DBH)

Here, a and b are species-specific parameters, DBH is the diameter at breast height, and H is the tree height.

**Table 3:** Variable characteristics.

Variable Type	Variable Name	Description	Formula
Spectral information	B2, B3, B4, B5, B6, B7, B8, B11, B12	Sentinel-2 image band information	
Vegetation index	NDVI	Normalized difference vegetation index	$\frac{B8-B4}{B8+B4}$
	RENDVI	Red-edge normalized difference vegetation index	$\frac{B6-B5}{B6+B5}$
	NDWI	Normalized difference water index	$\frac{B3-B8}{B3+B8}$
	EVI	Enhanced vegetation index	$\frac{2.5\times \left(B8-B4\right)}{B8+6\times B4-7.5\times B2+1}$
	RVI	Ratio vegetation index	$\frac{B8}{B4}$
	DVI	Difference vegetation index	B8−B4
	VDVI	Visible-band difference vegetation index	$\frac{2\times B3-B4-B2}{2\times B3+B4+B2}$
	SAVI	Soil-adjusted vegetation index	$\frac{1.5\times \left(B8-B4\right)}{B8+B4+0.5}$
	MSAVI	Modified soil-adjusted vegetation index	$\frac{\left[2\times B8+1-\sqrt{{\left(2\times B8+1\right)}^{2}-8\times \left(B8-B4\right)}\right]}{2}$
	NBR	Normalized burn ratio	$\frac{B8-B12}{B8+B12}$
	NDMI	Normalized difference moisture index	$\frac{B8-B11}{B8+B11}$
Tasseled cap transformation index	TCB	Tasseled cap brightness	$\begin{array}{l} 0.0356\times B1+0.0822\times B2+0.136\times B3+0.2611\times B4+\\ 0.2964\times B5+0.3338\times B6+0.3877\times B7+\\ 0.3895\times B8+0.0949\times B9+0.0009\times B10+\\ 0.3882\times B11+0.1366\times B12+0.475\times B8A \end{array}$
	TCG	Tasseled cap greenness	$\begin{array}{l} 0.0635\times B1+0.1128\times B2+0.168\times B3+0.348\times B4+\\ 0.3303\times B5+0.0852\times B6+0.3302\times B7+\\ 0.3165\times B8+0.0467\times B9+0.0009\times B10+\\ 0.4578\times B11+0.4064\times B12+0.3625\times B8A \end{array}$
	TCW	Tasseled cap wetness	$\begin{array}{l} 0.0649\times B1+0.1363\times B2+0.2802\times B3+0.3072\times B4+\\ 0.5288\times B5+0.1379\times B6+0.0001\times B7+\\ 0.0807\times B8+0.0302\times B9+0.003\times B10+\\ 0.4064\times B11+0.5602\times B12+0.1389\times B8A \end{array}$
Terrain information	DEM	Extract elevation information from the DEM	
	Aspect	Extract aspect information from the DEM	
	Slope	Extract slope information from the DEM	

B1-B12 and B8A are 13 spectral bands of Sentinel-2. B2, B3, B4, and B8 are blue, green, red, and near-infrared bands. B5, B6, and B7 are red-edge bands (red_edge1, red_edge2, and red_edge3), and B11 and B12 are short-wave infrared bands (swir1 and swir2).

**Table 4:** Tree height–DBH models’ fitting accuracy.

Tree Species	Model Number	R^2^	SSE	AIC
Poplar	1	0.81	454.94	170.20
	2	0.82	443.96	168.58
	3	0.77	568.19	203.05
	4	**0.82**	**432.91**	**162.85**
Mongolian Scots pine	1	**0.80**	**17.29**	**−118.55**
	2	0.78	18.24	−112.27
	3	0.74	22.37	−97.94
	4	0.80	17.36	−118.23
Chinese red pine	1	0.88	19.29	−134.62
	2	**0.88**	**19.23**	**−134.90**
	3	0.84	25.41	−109.82
	4	0.88	19.86	−132.00

## Data Availability

Data will be made available upon reasonable request.
